# Molecular and Biochemical Therapeutic Strategies for Duchenne Muscular Dystrophy

**DOI:** 10.3390/neurolint16040055

**Published:** 2024-07-05

**Authors:** Lakshmi Krishna, Akila Prashant, Yogish H. Kumar, Shasthara Paneyala, Siddaramappa J. Patil, Shobha Chikkavaddaragudi Ramachandra, Prashant Vishwanath

**Affiliations:** 1 Department of Biochemistry, JSS Medical College, JSS Academy of Higher Education & Research, Mysuru 570015, Karnataka, India; lakshmik@jssuni.edu.in (L.K.); akilaprashant@jssuni.edu.in (A.P.); shobhacr@jssuni.edu.in (S.C.R.); 2 Department of Medical Genetics, JSS Medical College, JSS Academy of Higher Education & Research, Mysuru 570015, Karnataka, India; 3 Department of Pharmaceutical Chemistry, JSS College of Pharmacy, Mysuru, JSS Academy of Higher Education & Research, Mysuru 570015, Karnataka, India; hyogishkumar@jssuni.edu.in; 4 Department of Neurology, JSS Medical College, JSS Academy of Higher Education & Research, Mysuru 570015, Karnataka, India; shasthara.p@gmail.com; 5 Department of Medical Genetics, Narayana Hrudalaya Health Hospital/Mazumdar Shah, Bengaluru 560099, Karnataka, India; drsjpatil@gmail.com

**Keywords:** muscular dystrophy Duchenne 1, CRISPR-Cas systems 2, CRISPR-Cas systems 3, histone deacetylase inhibitors (HDACis), drugs investigational

## Abstract

Significant progress has been achieved in understanding Duchenne muscular dystrophy (DMD) mechanisms and developing treatments to slow disease progression. This review article thoroughly assesses primary and secondary DMD therapies, focusing on innovative modalities. The primary therapy addresses the genetic abnormality causing DMD, specifically the absence or reduced expression of dystrophin. Gene replacement therapies, such as exon skipping, readthrough, and gene editing technologies, show promise in restoring dystrophin expression. Adeno-associated viruses (AAVs), a recent advancement in viral vector-based gene therapies, have shown encouraging results in preclinical and clinical studies. Secondary therapies aim to maintain muscle function and improve quality of life by mitigating DMD symptoms and complications. Glucocorticoid drugs like prednisone and deflazacort have proven effective in slowing disease progression and delaying loss of ambulation. Supportive treatments targeting calcium dysregulation, histone deacetylase, and redox imbalance are also crucial for preserving overall health and function. Additionally, the review includes a detailed table of ongoing and approved clinical trials for DMD, exploring various therapeutic approaches such as gene therapies, exon skipping drugs, utrophin modulators, anti-inflammatory agents, and novel compounds. This highlights the dynamic research field and ongoing efforts to develop effective DMD treatments.

## 1. Introduction

The dystrophinopathies, including Duchenne muscular dystrophy (DMD), Becker muscular dystrophy (BMD), and DMD-associated dilated cardiomyopathy (DCM), represent a spectrum of X-linked muscle disorders ranging from mild to severe. Mild phenotypes involve non-symptomatic elevation of serum creatine phosphokinase (CK) and myoglobinuria-associated muscular cramps, while severe cases include progressive muscle diseases like DMD-associated DCM, primarily affecting the heart, and Duchenne/Becker muscular dystrophy, primarily impacting skeletal muscle. (https://www.ncbi.nlm.nih.gov/gtr/conditions/C3668940/ accessed on 19 June 2024).

**Figure 1:**
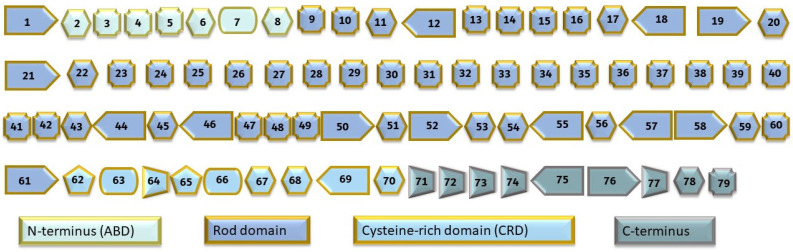
Exons of dystrophin gene. The DMD gene encodes 79 exons coding for the N-terminus ABD domain, rod domain, cysteine-rich domain (CRD), and C-terminus of dystrophin protein.

DMD typically manifests in early infancy with delayed motor milestones, such as difficulty rising from a supine position and walking independently. Proximal muscle weakness results in waddling gait and challenges in activities like sprinting, leaping, climbing stairs, and rising from a crouch. DMD progresses rapidly, often requiring wheelchair use by age 12 [1]. Cardiomyopathy usually develops after age 18, and most individuals do not live into their third decade, with cardiomyopathy and respiratory complications being the leading causes of death. BMD, characterized by later-onset muscle weakness, sees heart failure as the main cause of morbidity and mortality, with the average age of death in the mid-40s. DMD-associated DCM is marked by congestive cardiac failure and left ventricular dilatation [2]. Mutations in the DMD gene (Figure 1) cause DMD. This gene encodes the 3685 amino acid dystrophin protein, which connects the intracellular cytoskeleton to the dystrophin-associated protein complex (DAPC). Its four functional domains are the actin-binding amino-terminal domain (ABD), the central rod domain, the cysteine-rich domain (CRD), and the carboxy-terminal domain (CT). The absence of dystrophin leads to progressive muscle atrophy and weakening, affecting both skeletal and cardiac muscles [3]. Methods like the Multiple Ligation Probe Assay (MLPA) or microarray-based comparative genomic hybridization (array-CGH) detect deletions and duplications for molecular diagnosis [4,5]. Current standard treatment primarily aims to alleviate symptoms using glucocorticoids, although there is no cure for DMD. Contemporary therapeutic approaches are divided into dystrophin-targeted therapies (gene-based, cell-based, and protein replacement therapies) and strategies focusing on ameliorating muscle pathology caused by fibrosis, muscle atrophy, and calcium dysregulation [6].

This review summarizes key developments in both therapeutic areas, emphasizing those that have reached clinical trials or show promise for clinical application. It evaluates the efficacy and rationale of each treatment based on pre-clinical and clinical data, and provides a comprehensive overview of approved and ongoing clinical studies for DMD, highlighting ongoing efforts to develop effective treatments.

## 2. Possible Therapeutic Approaches

### 2.1. Pharmaceutical Methods That Attempt to Repair the DAPC and Focus on the Fundamental Problem

**Figure 2:**
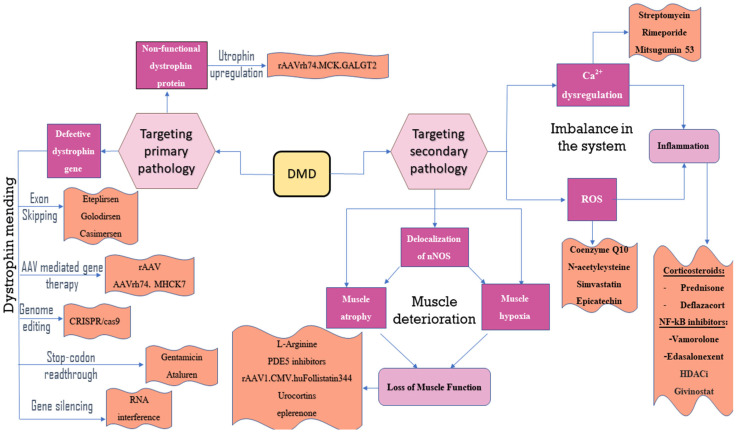
Potential therapeutic routes. Exon skipping, AAV gene editing, CRISPR-Cas9, stop codon readthrough, RNA interference, and utrophin modulation are a few promising therapeutic avenues. A secondary strategy involves small molecules that target the conditions that are associated with the primary complaints.

Despite extensive research efforts over many years, an absolute cure for DMD remains elusive; however, recent advancements in treatment provide renewed optimism. Several promising gene therapies are currently under investigation, including exon skipping, gene replacement, and stop codon suppression (Figure 2) [7]. Antisense oligonucleotides cause exon skipping at the molecular level. Presently, the use of adeno-associated viral vectors for the delivery of recombinant dystrophin genes is recommended for the development of gene therapies for DMD [8]. CRISPR-mediated deletions or excisions of intragenic DNA represent novel approaches for repairing the DMD gene, thereby restoring the expression of a truncated yet functional dystrophin protein [9].

#### 2.1.1. Exon Skipping

Exon skipping represents a promising therapeutic strategy for DMD, aiming to correct the altered open reading frame of dystrophin transcripts by omitting the mutant exon or adjacent exons. Three primary experimental approaches for exon skipping include antisense oligonucleotides (AONs), CRISPR-mediated DNA editing, and U7 small nuclear ribonucleoprotein (snRNP)-mediated splice blocking. Antisense oligonucleotides are chemically synthesized DNA-like molecules that complementarily bind to specific exons, preventing their splicing and inducing exon skipping along with nearby introns. The process of pre-mRNA splicing may skip exons surrounding a frameshift deletion, a key mechanism proposed for producing a functional dystrophin protein in DMD patients. Exon skipping AON therapy is mutation-specific, necessitating the development of multiple drugs to address the diverse mutations within the patient population. Among DMD patients, the five most commonly affected exons are exon 51 (accounting for 14% of all mutations or 21% of deletions), exon 53 (10%/15%), exon 45 (9%/13%), exon 44 (7%/11%), and exon 43 (7%/11%). Consequently, clinical trials have concentrated on evaluating drugs targeting these specific exons. For example, Eteplirsen and Drisapersen target exon 51, Golodirsen and Viltolarsen target exon 53, and Casimersen targets exon 45 [10]. An out-of-frame DMD deletion is intended to be changed into an in-frame deletion that resembles BMD (Becker Muscular Dystrophy) using CRISPR DNA editing techniques [6,11]. U7 snRNP260-mediated inhibition of splicing is a third method for achieving exon-skipping and has a similar mechanism of action to oligonucleotide methods. Adeno-associated viral (AAV) vectors have been employed as delivery vehicles for modified U7 snRNP genes. Unlike the usual antisense part that hybridizes to histone RNA, this section targets a dystrophin exon [12].

##### 2′-O-Methyl-Phosphorothioate (2′OMePS) Modification

**Figure 3:**
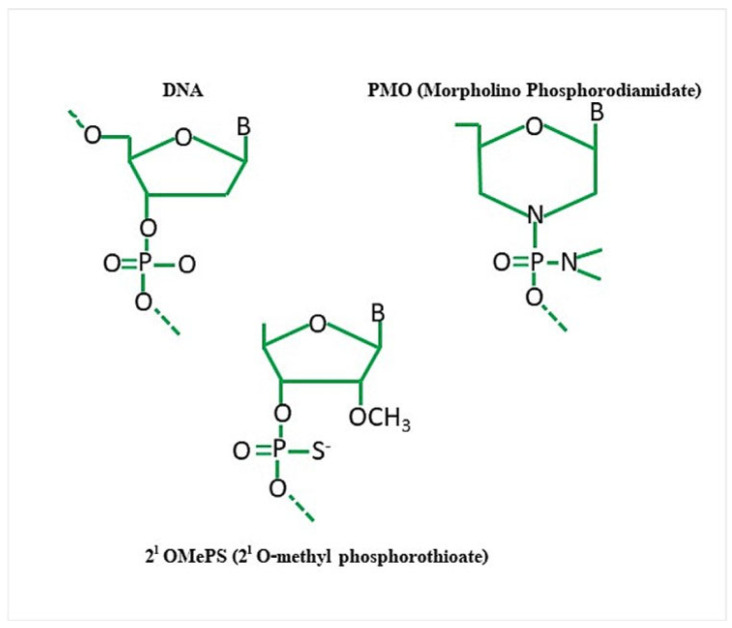
DNA and its analogs (antisense oligonucleotides (AONs)). 2′-O-Methyl-Phosphorothioate (2′OMePS), and morpholino phosphorodiamidate (PMO) are the extensively studied AONs.

AONs with distinct chemical attributes have been put together in order to give them nuclease resistance without compromising their capacity to hybridize with the proper target. The two AONs that have been the most extensively explored are 2′-O-Methyl-Phosphorothioate (2′OMePS) and morpholino phosphorodiamidate oligomers (PMOs) (Figure 3).

2′OMePS is an antisense oligonucleotide with a methylated c-2 position in the ribose ring of its phosphorothioate backbone [13]. Endonucleases are not able to immediately degrade the 2′OMePS as its phosphate group contains a sulfur atom in place of the nonbridging oxygen. Their complexing with cationic lipids and proteins is made possible by their negative charge, which also makes them more soluble [14]. Drisapersen and DS-5141b are proposed 2′OMePS compounds that, respectively, target exons 51 and 45 [13].

##### Phosphorodiamidate Morpholino Oligomer (PMO) Modification

PMOs, or phosphorodiamidate morpholino oligomers, are synthetic DNA analogs distinguished by a backbone composed of interconnected six-sided morpholine rings joined by phosphorodiamidate linkages. PMOs are neutrally charged, which improves tolerance by lowering immune reactions and off-target effects [14]. When compared to conventional synthetic oligonucleotides, PMOs demonstrate significantly less metabolic degradation and are substantially less sensitive to endo- and exonucleases. PMO-based drugs like Eteplirsen, Golodirsen, and Casimersen have been developed and have acquired conditional FDA approval for exon skipping of 51, 53, and 45 respectively.

- Eteplirsen

**Figure 4:**
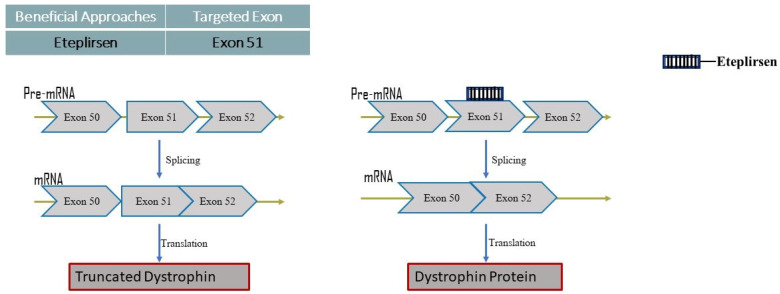
Exon (51) skipping by Eteplirsen. The dystrophin gene exon 51, which is frequently mutated in DMD, is the target of Eteplirsen. By omitting this exon, the gene’s reading frame is restored and a partially functional dystrophin protein can be produced.

Eteplirsen stimulates the production of dystrophin by directly acting on the DMD gene (Figure 4). Instead of the five-membered ribofuranosyl rings present in DNA and RNA, Eteplirsen contains a six-membered morpholino ring. In September 2016, the FDA granted its initial approval to Eteplirsen for the treatment of DMD in individuals with a confirmed mutation in the DMD gene that responds to exon 51 skipping [15].

Mode of action:

Eteplirsen does not exactly eliminate DMD, but Eteplirsen tries to alleviate the disease’s progression. On administration, Eteplirsen raises the levels of the dystrophin protein in individuals with DMD by generating mRNA for a shortened functional dystrophin protein. Thus, Eteplirsen helps to lessen the severity of the illness and slow down the rate of functional deterioration. Eteplirsen binds to exon 51 of the dystrophin pre-mRNA in patients where exon 51 is present but part of a larger deletion mutation that disrupts the reading frame. By skipping exon 51 during mRNA processing, Eteplirsen can re-establish the open reading frame, enabling the production of a functional dystrophin protein. This strategy is particularly useful in cases where exon 51 is not itself deleted but is part of a disrupted sequence. (https://go.drugbank.com/drugs/DB15593) accessed on 5 May 2023.

- Golodirsen

**Figure 5:**
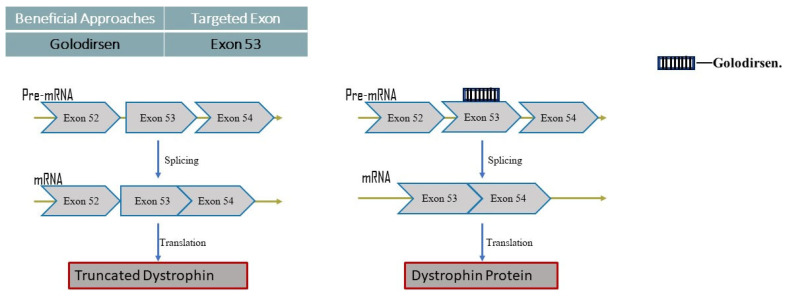
Exon (53) skipping by Golodirsen. Golodirsen targets exon 53 of the dystrophin gene. It functions by attaching to exon 53 and preventing the inclusion of that region in the final mRNA transcript. As a result, a functional but shortened dystrophin protein is produced, which can help DMD patients’ muscles function better.

Another morpholino antisense oligomer called Golodirsen is intended to treat around 8% of people with DMD. On 12 December 2019, the FDA granted fast approval to the medicine produced by Sarepta Therapeutics since DMD sufferers urgently needed it. Although clinical trials did not detect any appreciable damage, Golodirsen’s FDA approval was initially denied due to worries about its potential renal toxicity (Figure 5) [16].

Mode of action:

In order to treat DMD, Golodirsen conceals genetic abnormalities that set off a chain of events. Exon 53 of the dystrophin pre-mRNA on the DMD gene is blocked by Golodirsen, which prevents this protein coding region from being processed. Dystrophin is produced as a result of the exclusion (or skipping) of exon 53 by Golodirsen by converting out-of-frame mRNA to in-frame mRNA. BMD, a less severe disorder marked by the creation of a shortened dystrophin protein, is apparently induced by the development of an incomplete dystrophin protein driven by Golodirsen. In general, patients with BMD can anticipate a longer lifetime and higher quality of life. (https://go.drugbank.com/drugs/DB15593) accessed on 5 May 2023.

- Casimersen

**Figure 6:**
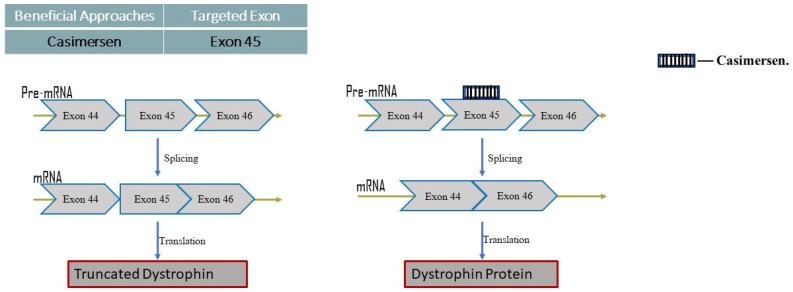
Exon (45) skipping mechanism by Casimersen. An illustration of Casimersen promoting exon (45) skipping in the dystrophin gene to carry out its function. Exon skipping occurs during mRNA processing because the medication binds to a specific region in the targeted exon that prevents the binding of splicing components. The resultant mRNA is devoid of the mutant exon, which fixes the reading frame and results in the synthesis of a dystrophin protein that is only partially functional.

The most recently accepted PMO for the treatment of DMD is Casimersen (Figure 6).

Mode of action:

Patients with mutations that can be treated with exon 45 skipping are given this antisense phosphorodiamidate morpholino oligonucleotide. In order to produce an internally shortened but functional dystrophin protein in DMD patients, Casimersen is made to bind to exon 45 of the DMD gene pre-mRNA. This skips this exon 45 during mRNA processing [17].

##### AAV-Mediated Gene Therapy (Adeno-Associated Viral Vector Delivery)

The dystrophin cDNA size is approximately 14 kb, whereas AAV can accommodate approximately 4.5 kb of genetic material for cloning. The dystrophin cDNA is split over two AAV cassettes to enable the delivery of larger, potential mini dystrophins, with the 5’ and 3’ ends joined through homologous recombination or trans-splicing. These microdystrophins lack half of the typical dystrophin amino acid sequence and are biochemically similar yet smaller than Becker-like dystrophins that are naturally found in BMD patients [18]. However, these microdystrophin genes encode proteins that are functional yet shortened, as they lack extraneous regions including most of the rod domain and the distal C-terminal domain. In comparison to untreated patients, the outcome is a weaker dystrophic phenotype. Several varieties of microdystrophin were given to mdx mice either locally or systemically, and the treatment reduced disease pathology and enhanced muscular performance. Recombination of two overlapping (ov) rAAV vectors and trans-splicing of two vectors (ts) have both been investigated as ways to increase the AAV vector’s packaging capacity. In skeletal muscle, a composite dual-vector system combining the benefits of the ts and ov vectors has demonstrated effectiveness. Preclinical investigations conducted on dystrophic canines have focused on the enhancement of pathology and muscle function resulting from successful AAV-mediated gene transfer into dystrophic mice. Transient immune suppression was required in dogs because significant cellular immunological responses were frequently seen. However, it is unclear why rAAVs cause dogs to have significantly more powerful immunological reactions than mdx mice [19]. The ability of codon optimization to lower virus titers has also been established. AAV2.5-CMV-MiniDystrophin was used to administer microdystrophin intramuscularly in a Phase 1/2 clinical trial in 2006 (PI: JR Mendell; Trial ID: US-679; clinicaltrials.gov identifier: NCT00428935). http://www.clinicaltrials.gov accessed on 8 May 2023. Three gene therapy programs for DMD are now accepting new participants. Each one makes use of a muscle-specific promoter that controls the expression of several microdystrophins. The AAVrh74 capsid with the MHCK7 promoter is what the Sarepta program employs. SGT-001, developed by Solid Biosciences in Cambridge, MA, utilizes an AAV9 capsid to express microdystrophin under the control of the CK8 promoter. Similarly, PF-06939936, developed by Pfizer in New York, NY, employs an AAV9 capsid coupled with a muscle-specific promoter for its function [20].

One more study is underway, the NCT05429372 trial, which will evaluate dystrophin expression and safety in boys with DMD after receiving gene therapy. This trial is an open-label, single-arm, non-randomized study aimed at assessing the tolerance and safety of fordadistrogene movaparvovec gene therapy. There will be no placebo arm in this trial, and approximately ten participants will be recruited to receive a single IV (intravenous) infusion of PF-06939926. To be eligible for the trial, all males must test negative for neutralizing antibodies against AAV9 as part of the screening process. Following gene therapy treatments, all participants in the trial will be monitored for a period of five years (https://clinicaltrials.gov/study/NCT05429372) accessed on 8 May 23.

The advantages of AAV vectors are epi-chromosomal retention in the host cell, significant transduction effectiveness in both dormant and proliferating cells, and broad tropism for many tissue targets [21]. The AAVs are divided into more than 10 serotypes depending on capsid proteins, each of which exhibits a particular cell morphology. Many AAV serotypes, namely rAAV1, rAAV2, rAAV5, rAAV6, rAAV7, rAAV8, and rAAV9, have demonstrated effective muscle transduction. AAV8 and AAV9 are the best treatments for extensive muscle diseases that affect the heart and diaphragm because they can effectively reach many muscles, which are challenging to access with local injections [22].

The replacement of the defective gene (gene replacement), in comparison to exon skipping, is not mutation-specific. Despite its accessibility and profusion (30–40% of the body), delivering genes to the muscle is difficult. As a result, plasmid-based gene delivery is less effective. Moreover, aside from AAV, the majority of viral vectors are ineffective at transducing muscle. The vector must be delivered systemically in order to reach all of these muscles. Because of the enormous doses needed, the safety profile of the chosen vector becomes even more crucial. The choice of a promoter that exhibits high levels in skeletal and cardiac muscle and low levels in off-target organs is also required due to the emphasis on these tissues. Transgene expression is limited due to the implementation of tissue-specific promoters [23]. Testing of systemic AAV delivery in canines, non-human primates, and patients has been performed. These studies’ findings revealed a number of drawbacks, including (1) inconsistent AAV delivery to the intended limb and (2) inability to access the heart and diaphragm, two of the most important targets [24]. Overcoming immunity and delivery are also the two biggest obstacles to AAV-mediated gene therapy. Another major risk with rAAV-mediated gene therapy for DMD is immunity to AAVs. First, naturally occurring AAV infections are highly prevalent in human populations, and pre-existing antibodies may prevent the use of AAV vector-mediated therapy. Second, a neutralizing antibody is known to make the second injection of rAAV vectors far less effective after the initial injection. Indeed, according to clinical studies involving AAV vectors, the main drawback of rAAV-mediated gene therapy is the immunological response to the vector and/or transgenic product. Utilizing a muscle-specific promoter that is active in both skeletal and cardiac muscles is favored to minimize host immunological reactions against the transgenic product [25]. Currently, ongoing clinical investigations are evaluating the systemic administration of various microdystrophin cDNA variants using different serotypes of AAV vectors at significant dosages (1–3 × 10^14^ vectors/kg). It is unclear if this treatment can slow disease progression, but early findings from these studies suggest that 80% of muscle fibers have detectable microdystrophin. However, notable side effects, including acute renal failure potentially triggered by an innate immune response and temporary elevation of liver enzymes due to a possible cellular immune reaction to the vector, have been observed in a subset of patients [1]. Despite the increasing number of clinical trials employing rAAVs, there are still numerous challenges yet to be addressed in this field. The primary obstacle to AAV-based gene therapies remains the immunogenicity of the vector, posing a significant challenge for gene therapy approaches. Thus far, decreasing efficacy has primarily been attributed to adaptive immunity targeting the capsid and the foreign transgene. Nonetheless, the need for repeated dosing may be mitigated by the single-injection feature and the long-lasting effects of AAV gene therapy [26].

##### CRISPR-Cas9 Genome Editing

**Figure 7:**
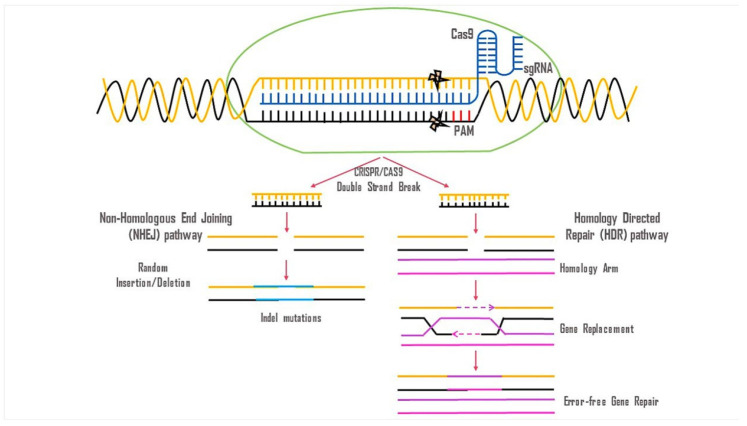
Mechanism of CRISPR/Cas-9 genome. Specific double-stranded DNA breaks can be introduced into the genome via CRISPR/Cas9 technology, which subsequently triggers DNA repair processes. And the DNA repair mechanisms will correct missense mutations through non-homologous end joining (NHEJ)-based prenatal editing or homology-directed repair (HDR)-based germline editing.

Genome editing is a form of genetic engineering in which DNA is deliberately added, deleted, or altered in living cells. The development of Clustered Regularly Interspaced Short Palindromic Repeats (CRISPR) and CRISPR-associated (Cas) technology has significantly expanded the scope of genome editing, establishing it as a pioneering tool in efficient genomic engineering. CRISPR/Cas9, originally identified as part of the bacterial adaptive immune system’s defense against bacteriophages, facilitates precise genome editing by creating specific double-stranded DNA breaks. These breaks activate DNA repair mechanisms such as homology-directed repair (HDR) or non-homologous end joining (NHEJ) to correct mutations (Figure 7) [27]. Exon skipping at the DNA level, facilitated by antisense oligonucleotides and CRISPR-mediated deletions or excisions of intragenic DNA, represents promising strategies for repairing the DMD gene, thereby restoring the expression of a truncated yet functional dystrophin protein. Other CRISPR/Cas9-based approaches for treating DMD include the removal of duplicate exons, precise repair of the causal mutation via HDR, and the induction of compensatory protein production, such as utrophin [9]. Compared to exon skipping therapies, CRISPR/Cas9 offers several advantages, including the potential to treat patients with duplications in specific exons of the DMD gene and the possibility of eliminating the need for repeated administrations, as DNA, rather than pre-mRNA, is targeted [28].

To administer CRISPR/Cas9 in vivo, AAVs have become a popular choice. Utilizing AAV8-CRISPR/Cas9 therapy, it is possible to promote the generation of functional dystrophin protein within skeletal muscle fibers. In studies involving mdx mice with exon 23 deletions, this approach has shown promising results, significantly increasing muscular force [29]. AAV6-CRISPR/Cas9 can also considerably boost muscle strength in mdx mice and fix both exon 52 and exon 53 deletions, according to a separate study [30]. Clinical benefits from CRISPR/Cas9 gene editing delivered by AAV vectors may be observed in around 60% of DMD patients. The effectiveness of CRISPR/Cas9-mediated genome editing is heavily reliant on the dosage of AAV utilized. Likely, exogenous AAVs and Cas9 will not work well in therapeutic situations due to the strong immune responses they elicit. Studies have shown that dystrophin and the protein complex are related and that CRISPR-mediated gene editing can restore them at the sarcolemma [31].

The CRISPR/Cas-9 genome editing system consists of three stages: recognition, cleavage, and repair. The targeted sgRNA (single-guide RNA) recognizes the target sequence in the respective gene by its complementary base pair in the 5′ crRNA (CRISPR RNA), which governs Cas-9. The Cas-9 protein stays inactive in the absence of sgRNA. The Cas-9 nuclease causes double-strand breaks (DSBs) to three base pairs upstream of PAM (Protospacer Adjacent Motif). The PAM sequence is a short, conserved sequence of DNA that is situated downstream of the cut spot. It ranges in length from two to five base pairs. The most widely used nuclease in genome editing tools, the Cas-9 enzyme, can recognize the PAM sequence at 5′-NGG-3′ (where N can be any nucleotide base). However, it is still unclear exactly how the Cas-9 enzyme melts the target DNA sequence. Target DNA is predominantly broken up into blunt-ended double-strand breaks (DSBs) by the HNH and RuvC domains, respectively, into its complementary and non-complementary strands. In the end, the DSB is fixed by the host cellular machinery. In the absence of external homologous DNA, NHEJ functions as an enzymatic method to join DNA fragments, assisting in the repair of double-strand breaks (DSBs). This mechanism is active across the whole cell cycle. HDR is incredibly accurate, although it does need the aid of a homologous DNA template. The cell cycle is particularly active in the late S and G2 stages (Figure 7) [32,33].

The CRISPR/Cas9 genome editing system’s ease of use and adaptability have accelerated its spread and adoption. It has demonstrated remarkable robustness in altering gene sequences in human cells, paving the way for new opportunities such as the utilization of efficient multiplex gene editing for the simultaneous deactivation of multiple genes. CRISPR/Cas9 can alter gene sequences precisely and permanently, with modifications that last after cell division. Furthermore, unlike exon skipping, which requires a unique oligonucleotide to be constructed for each exon to be eliminated from the mRNA transcript, only two nucleases are required to delete a genomic area of any length. It has been suggested that restoring the native dystrophin gene in the cells of DMD patients can be accomplished through genome editing employing a variety of designer nucleases. Developing numerous nucleases specifically targeted to exons and introns, necessary to treat a significant portion of the DMD patient population, has posed a challenge. However, the protein product resulting from the altered gene is predictable and has already been characterized in BMD individuals with the naturally occurring deletion, which represents another advantage of the current technology over the indel-based approach. The usefulness of this technique for repairing the dystrophin gene and generating other specific genetic alterations will expand with further enhancements in the delivery, specificity, and efficacy of these reagents. Consequently, CRISPR/Cas9 genome editing presents a novel and interesting route for the development of gene treatments for DMD [34,35].

##### Stop Codon Readthrough

**Figure 8:**
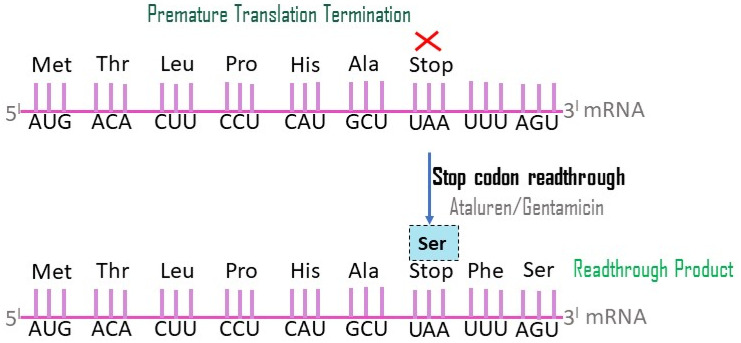
Stop codon readthrough. Stop codon readthrough illustrated schematically. Stop codon readthrough takes place when a premature stop codon in the messenger RNA is neglected, allowing translation to go on and producing a functioning dystrophin protein.

Stop codon readthrough, which allows translational machinery to bypass the premature stop codon caused by nonsense mutations in the dystrophin gene, is a potentially effective treatment for DMD. Around 10–15% of DMD mutations involve premature stop codons, which are specific sequences of mRNA nucleotides that interfere with ribosomal function, resulting in the early termination of protein translation. As a result, a working dystrophin protein is not synthesized. By attaching to the ribosomes and preventing the DMD cell lines from recognizing the stop signals, nonsense suppression therapy or stop codon therapy causes readthrough of these premature stop codons, resulting in the production of a full-length modified dystrophin protein. Thus, using this strategy, 10–15% of all DMD patients can be treated. It is suitable for all nonsense mutations in DMD (Figure 8) [36].

- Gentamicin

**Figure 9:**
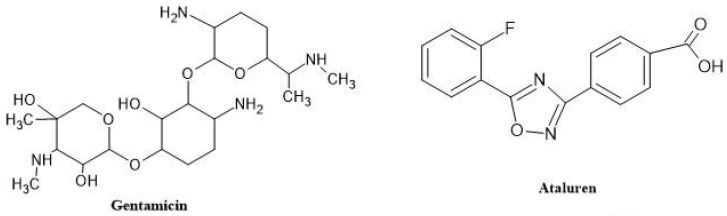
Gentamicin and Ataluren structure.

When gentamicin (Figure 9) detects a stop codon, it engages the translational apparatus (40S ribosomal subunit). This process results in the insertion of an amino acid at the stop codons found within the mRNA, allowing the translational machinery to proceed with mRNA translation. It specifically occurs in premature stop codons because normal stop codons and nonsense mutations are separated by different nucleotide sequences. Gentamicin has been tested as a DMD therapy. This drug resulted in up to 20% of fibers being dystrophin-positive when given to dystrophic mdx mice. This hopeful discovery led to the beginning of two clinical trials including patients with DMD and BMD [37].

Gentamicin can disrupt the area of the ribosome that tracks connections between codons and their anticodons with nearby aminoacyl-transfer RNAs (tRNAs) or suppressor tRNAs. The PTC (premature termination codon) gets replaced with another amino acid as a result of this interaction. In prokaryotic cells like bacteria, aminoglycosides exhibit a strong affinity for the 16S rRNA subunit. This affinity induces a conformational change akin to that caused by homologous tRNA binding. Consequently, there is considerable misinterpretation of both sense and nonsense codons, leading to aberrant expression patterns and cell death. However, due to structural disparities between eukaryotic and bacterial cells—specifically in their 18S and 16S rRNA components, respectively—the affinity of aminoglycosides for eukaryotic cells is much lower. Consequently, the likelihood of misincorporation at premature termination codon (PTC) sites is significantly reduced [38].

Notably, while readthrough of standard stop codons has not been observed in eukaryotic cells with any of the antibiotics studied thus far, the substitution of a stop codon with an amino acid appears to be unique to PTCs. This specificity and selectivity may be attributed to the intricate transcription machinery in eukaryotic cells, which includes multiple proteins and regulatory elements governing the normal termination of protein synthesis, ensuring that the protein halts at its conventional stop codon. Furthermore, the efficacy and accuracy of aminoglycosides’ readthrough activity depend on the type of stop codon targeted [39].

Gentamicin was tested in two different animal models of DMD—first in a cell line from people with cystic fibrosis, and then in mdx mice, who are lucky carriers of the disease’s UAA premature stop mutation. This mdx proof-of-concept study revealed that dystrophin was correctly localized to the sarcolemma and that expression of muscle fiber dystrophin was 20% normal in vitro and in vivo. The muscle fibers demonstrated heightened resistance to damage from eccentric contraction. Creatine kinase leakage from muscle into blood was found to be lessened, which may indicate less brittle muscle cells. These results served as the foundation for human trials of intravenous gentamicin. Between 2001 and 2003, there were two brief pilot studies on the use of gentamicin in DMD patients with nonsense mutations. Wagner et al. carried out the initial study. In this investigation, a regimen of daily intravenous administration of gentamicin at a dosage of 7.5 mg/kg was employed over a period of 2 weeks in four individuals (two diagnosed with DMD and two with BMD), aged between 7 and 16 years. Despite this treatment, there was no observed drug activity throughout the brief duration of drug exposure, as evidenced by the lack of discernible changes in muscle dystrophin expression upon biopsy analysis, as well as in muscle strength. Notably, no renal or ototoxic adverse effects were observed during the course of the study [40]. The effects of intravenous gentamicin treatment in four DMD children, aged 4 to 9 years, were described by Politano et al. in 2003. Three of the subjects were ambulant, while one used a wheelchair. They employed a gentamicin regimen that included two six-day rounds of treatment at doses of 6.0 and 7.5 mg/kg, respectively, separated by a seven-week break. In end-of-treatment biopsies, they showed a rise in dystrophin expression in three of four individuals, with the youngest patient seeing the best outcomes. Furthermore, they exhibited re-expression of the sarcoglycan complex, indicating a restoration of sarcolemmal integrity. Importantly, there were no observed instances of ototoxicity or renal toxicity during the study period [41]. However, several aspects have to be kept in mind when utilizing gentamicin for the treatment of DMD. The dose required to produce appropriate dystrophin expression and the dose that can result in renal damage and ototoxicity is separated by a small therapeutic index. Second, the burden of the treatment is increased by the requirement for routine intravenous administration, evaluation of drug levels, and quality laboratory data. Finally, the ability of different gentamicin forms to stimulate dystrophin expression varies considerably [42].

- Ataluren

Ataluren (PTC124/Translarna) **(**Figure 9) is a new, orally administered drug that targets nonsense mutations and reads through premature termination codons (UGA, UAG, and UAA) during mRNA transcription, theoretically allowing the expression of fully functional dystrophin protein and avoiding aminoglycosides’ potential renal toxicity and ototoxicity. (https://go.drugbank.com/drugs/DB15593) accessed on 23 May 2022.

Ataluren prevents premature protein chain termination by allowing ribosomal readthrough of mRNA with premature stop codons. By using ataluren, cellular machinery can continue translating genetic material despite nonsense mutations, restoring the synthesis of a fully functional protein. According to studies on the impact of ataluren on the stability and translation of nonsense codon-containing mRNA in vitro, ataluren enhanced readthrough at all nonsense codons, with UGA demonstrating the highest level of activity while having no impact on mRNA levels. Ataluren did not distinguish between the UAG and UAA mRNAs appreciably, in contrast to the stable cell line experiments. At levels similar to those observed in the stable cell reporter experiments, ataluren exhibited a 4- to 15-fold increase in in vitro readthrough compared to the control group, indicating its superior efficacy as a nonsense-suppressing drug compared to gentamicin. These findings suggest that ataluren modifies the efficacy of termination at premature nonsense codons. (https://go.drugbank.com/drugs/DB15593) accessed on 23 May 2022.

Treatment with PTC124 in trials using mdx mice has demonstrated promise in resuming dystrophin synthesis in skeletal muscles, including the diaphragm and heart muscle. Dystrophin (20–25%) was present in treated mice compared to mouse muscles from control animals. A decrease in muscle fragility was also shown by the treatment group’s increased resistance to eccentric exercise and partial restoration of muscular function. Sarcoglycan levels rose after PTC124 treatment, indicating that dystrophin-associated proteins may have stabilized. Within 2 to 8 weeks of the start of the treatment, the beneficial effects on skeletal muscle were noticeable. PTC124, which did not impair normal stop codon readthrough, showed selective and specific readthrough of disease-causing premature stop codons. The beginning of additional human trials was motivated by these optimistic preclinical findings [41].

Thirty-eight boys with DMD with the nonsense mutation were enrolled in a Phase 2a open-label, dose-ranging trial; participants were segmented into three groups according to the dosage of Ataluren administered: 16 mg/kg/day (6 patients), 40 mg/kg/day (20 patients), and 80 mg/kg/day (12 patients). The duration of treatment spanned 28 days. The primary outcome was the difference in full-length dystrophin expression between pre-treatment and post-treatment muscle biopsy samples as determined by immunostaining. They discovered that after therapy, dystrophin expression increased in 61% of the cases and was not related to the exon location or kind of nonsense mutation. Based on these proof-of-concept findings, a Phase 2b trial with 48 weeks of ataluren therapy was set up to assess the safety and effectiveness in DMD and BMD patients with a nonsense mutation. At 37 sites, 174 people were enrolled in the study. The primary endpoint in this trial was the 6 min walk test, supplemented by evaluations of muscular function and strength, as well as analysis of muscle dystrophin expression in biopsies taken before and after treatment. PTC Therapeutics announced preliminary findings after the study’s intake and therapy phases were complete. Over the course of the 48 weeks of therapy, there was an extremely high rate of drug compliance and no serious safety concerns were found. There was no discernible change between the treated groups (40 mg/kg/day and 80 mg/kg/day) and the placebo group for the 6 min walk distance. Additional investigation into patient groups, muscle dystrophin expression, exon position, and nonsense mutation type is still awaited [42].

The European Medicines Agency has approved this drug for the treatment of DMD in patients who can walk and are at least 5 years old. Nevertheless, neither the US Food and Drug Administration nor Health Canada has yet approved this medication for any indications. (https://go.drugbank.com/drugs/DB15593) accessed on 23 May 2022.

##### Post-Transcriptional Gene Silencing/RNA Interference

Although not widely employed, RNA interference-based techniques have been discussed in previous research and even been put to the test in clinical studies. A combined therapy, which involved activin receptor type IIb (ActRIIB), a myostatin receptor, or the soluble form of this receptor, and exon skipping to upregulate dystrophin, was evaluated on dystrophic animals. It has been demonstrated that myostatin, also known as growth differentiating factor 8 (GDF-8), a member of the superfamily of TGF-signaling molecules that acts through certain transmembrane receptors, primarily ActRIIB, contributes to DMD by regulating the growth and differentiation of muscle cells [43]. Inhibiting myostatin or ActRIIB using a variety of techniques (including RNA interference, exon skipping, antibodies against myostatin or ActRIIB, dominant-negative myostatin or ActRIIB, pharmacological suppression, etc.) has been shown to increase muscle mass and function in both dystrophic mdx mice and mice with wild-type muscles [44]. On the contrary, some studies have failed to observe any improvement in human subjects following the inhibition of myostatin/ActRIIB signaling, thereby casting doubt on the effectiveness of this therapeutic approach. Particularly noteworthy is the suspension of two clinical trials employing ACE-031, a soluble variant of human ActRIIB, due to initial safety concerns. Similarly, the myostatin antibody trials (conducted by Roche—NCT03039686 and Pfizer—NCT02907619) similarly fell short of the primary endpoint, casting doubt on the efficacy of this approach for DMD patients [45]. However, according to a recent study by Mariot et al., the low myostatin levels found in DMD patients may be to blame for the poor efficacy of anti-myostatin treatments. It was shown that numerous neuromuscular illnesses were associated with low levels of circulating myostatin and decreased myostatin pathway mRNA in muscle samples. Additionally, a study revealed that DMD patients presenting with cardiomyopathy exhibited significantly lower levels of myostatin compared to those without cardiac symptoms when assessing protein levels in both patient groups. As a result, the proposition of inhibiting this pathway through exogenous means such as monoclonal antibody treatment or vector-mediated suppression failed to yield improvements in phenotype, severely limiting the clinical utility of this approach. It is noteworthy that mdx mice demonstrate myostatin levels at least 50 times higher than those observed in individuals with DMD. This discrepancy may contribute to variations in the reported efficacy of anti-myostatin therapy between mouse models and human subjects [45].

##### Utrophin Modulation

Utrophin, a protein comprised of 3433 amino acids, has an approximate molecular weight of 400 kDa. It is also known as dystrophin-related protein (DRP). It has a significant degree of similarity with dystrophin and it can recruit the majority of the DAPC proteins. Utrophin is expressed heavily when dystrophin is absent. With 80% homology, the structures of the two proteins are quite similar. Both dystrophin and utrophin have rod domains in their structures, although both molecules differ significantly in the sequencing of this domain. Although dystrophin is present across the sarcolemma of muscle fibers, utrophin exhibits distinctive expression and localization limited to neuromuscular and myotendinous junctions within muscles [6]. Utrophin A and B are the only two fully developed isoforms of utrophin that have been discovered so far. From two separate promoters, A and B, these isoforms are translated. Two identical functional proteins with two slightly different N-terminal domains and two different expression patterns are produced as a result of the two mRNAs’ differences at their 5′ ends. Utrophin B is exclusively expressed in endothelial cells, unlike utrophin A, which is present in various tissues such as neuromuscular junctions, the choroid plexus, the pia mater, and the renal glomerulus. Intriguingly, recent discoveries have revealed five new 5′ utrophin isoforms (A′, B′, C, D, and F) in both human adult and embryonic tissues. Nevertheless, they have not yet been thoroughly described [46].

The generation of transgenic mice that exhibit utrophin overexpression provides evidence supporting the notion that utrophin has the potential to compensate for the lack of dystrophin in DMD. These transgenic mice further demonstrate the importance of utrophin’s continuous location along the sarcolemma and the potential benefits of modest (1.5-fold) utrophin increases. In contrast to the usual amounts found in the kidney and liver, the high level of utrophin shown in transgenic mice is far lower and is not hazardous in a variety of mouse tissues [47]. Hence, an efficient therapeutic approach for DMD entails utilizing small molecules to regulate utrophin levels via modulation of the promoter region. In the mdx mouse, it has been demonstrated that such tiny compounds can stop the disease. Ezutromid, formerly known as SMT C1100, has successfully finished Phase 1a and 1b clinical trials, demonstrating that it is well tolerated [48,49,50].

According to data from clinicaltrial.gov, a Phase 2 trial with this medication (NCT02858362) was halted because it did not demonstrate effectiveness. Summit Therapeutics has pursued the development of alternative molecules, albeit within the same class as ezutromid, demonstrating positive effects in mdx animal models. The ezutromid medication series and this method of utrophin regulation have been validated by other compounds created in identical chemical series that exhibit efficacy in mdx mice. Other medications, including AICAR (5-Aminoimidazole-4-carboxamide ribonucleotide), nabumetone, heregulin, and resveratrol, have exhibited impacts on the utrophin gene and demonstrated effectiveness in mdx mice. However, clinical studies for DMD involving these medications have not been reported [51]. The use of related pharmaceutical drugs to boost utrophin expression has been investigated. Utrophin expression is controlled by numerous processes including transcription, post-transcription, and other controls. Utrophin levels are increased by heregulin through the epigenetic control of the utrophin-A promoter, which is accomplished by activating mitogen- and stress-activated protein kinase (MSK1/2) and phosphorylating histone H3 in an ERK-dependent way [52]. By encouraging the slow oxidative phenotype, utrophin can also be raised. The elevation of utrophin and functional advantages are brought about by treatment with AICAR that targets the (PPAR-β/δ), 5′ adenosine monophosphate-activated protein kinase (AMPK), and sirtuin 1 (SIRT1) acting on the PGC-1(Peroxisome Proliferator-Activated Receptor Gamma Coactivator 1) levels [53]. Agonists of the PPAR-β/δ, like GW501516, also encourage the production of utrophin A and the oxidative phenotype. Heparin facilitates the retention of the RNA-binding protein KSRP (KH-type splicing regulatory protein) by the regulatory protein 14-3-3. This interaction leads to the post-transcriptional stabilization of utrophin mRNA. This is accomplished by activating p38 MAP Kinase [54,55]. New strategies for enhancing utrophin expression are being explored. One approach involves the use of oligonucleotides targeting the utrophin 3′UTR to inhibit let-7c binding, thereby increasing utrophin production. Mishra et al. investigated the effectiveness of oligonucleotides comprised of 2′-O-methylated nucleotides on a phosphorothioate backbone, termed let7-SBOs (Small Base modified oligonucleotides), using mdx mice as a model. Following one month of treatment via intraperitoneal injections, 2-month-old mdx mice exhibited elevated utrophin expression in the diaphragm, gastrocnemius, and tibialis anterior muscles. Assessment of muscle morphology and physiology, including muscle weight, muscle damage, inflammatory cell infiltration, and fibrosis, indicated improvement in the dystrophic phenotype upon let7-SBO administration [56]. Notably, the administration of utrophin may offer advantages over large doses of the AAV-mini-dystrophin gene, potentially reducing the risk of immune responses observed in canine models of DMD and GRMD (Golden Retriever model for DMD). The efficiency of microdystrophin and miniaturized utrophin supplied by AAVs were compared in an array of experiments by Song et al. In addition to preventing muscle damage in newborn mdx mice, it was shown that neonatal GRMD dogs lacking the full dystrophin gene did not have an immunological response and that adult dogs lacking the gene did not exhibit dystrophic symptoms [57]. At Nationwide Children’s Hospital, a different strategy is being implemented to control utrophin expression (NCT03333590). The goal of this work is to transfer the GALGT2 gene by adeno-associated virus (rAAVrh74.MCK.GALGT2) to cells, where it will be overexpressed. GALGT2 is recognized for its role in attaching the terminal GalNAc to an O-linked carbohydrate antigen located on α-dystroglycan, particularly prominent at the neuromuscular junctions. When GALGT2 is overexpressed in mdx mice, utrophin is expressed more frequently, which significantly improves the muscular membrane’s resilience to damage. Even if utrophin acts favorably to make up for dystrophin, it nevertheless performs some activities differently from dystrophin. As a result, it would be beneficial to consider treating DMD with a mix of utrophin-based medicines and other dystrophin-targeted medications [58]. The results were expected in October of 2023. The nitric oxide synthase (NOS) substrate l-arginine also promotes the upregulation of utrophin by increasing the generation of nitric oxide (NO), which suppresses the proteolytic activity of calpain [59]. Finally, some substances work by stabilizing utrophin at the sarcolemma. For example, biglycan, a small leucine-rich proteoglycan (SLRP), directs the assembly of a complex that is associated with utrophin and includes nNOS, increasing utrophin levels and significantly improving muscle function in mdx mice [60]. Sarcospan overexpression activates Akt and raises the amount of GALGT2, which in turn causes α-dystroglycan (α-DG) glycosylation. Enhancing the transit of utrophin-α-DG from the endoplasmic reticulum/Golgi membranes enhances utrophin expression on the cell surface. The utrophin protein complex is known to be stabilized by overexpression of CT-GalNAc transferase [61]. Thrombospondin-4 enhances the vesicular transport of dystrophin–glycoprotein and integrin attachment complexes, thereby promoting the stabilization of sarcolemmal proteins like utrophin [62]. The combination of agents aimed at stimulating utrophin expression through these pathways may offer cumulative benefits. Regulating utrophin expression shows promise as a potential treatment strategy for all individuals affected by DMD, regardless of the specific mutation in the dystrophin gene. The critical protective role of utrophin is indicated by milder disease manifestations observed in mdx mice expressing utrophin, although disease progression can worsen in DMD individuals with naturally elevated utrophin levels. However, it is important to note that utrophin lacks a NO binding site and may not fully mitigate functional ischemia during muscle contraction, thus limiting its ability to completely reverse the manifestations of dystrophin deficiency [63].

### 2.2. Drug Therapies That Focus on the Secondary Disease That Results from Dystrophin Deficiency

#### 2.2.1. Corticosteroids

Corticosteroids are recognized for enhancing muscular strength and delaying the loss of ambulation in individuals with DMD). However, their use remained highly variable until the publication of the first international guidelines for DMD therapy. Corticosteroids have demonstrated efficacy in slowing disease progression and extending functional capacity for two years or longer; thus, they are now considered the primary treatment for DMD. The precise mechanisms by which corticosteroids confer these benefits remain unclear. Additionally, evidence suggests that corticosteroids reduce the frequency of scoliosis surgeries and delay the onset of cardiomyopathy [64]. Prolonged and continuous administration of corticosteroids is associated with various adverse effects, including weight gain, osteopenia (exacerbated in DMD due to decreased bone loading), Cushingoid features, and delayed puberty. Given the well-documented risks of prolonged glucocorticoid therapy, the primary goal of administering glucocorticoids to ambulatory children is to preserve ambulation and mitigate the development of subsequent respiratory, cardiac, and orthopedic complications. Despite numerous clinical trials, uncertainties persist regarding the optimal timing for initiating and discontinuing treatment. Preclinical models are just beginning to elucidate the mechanisms underlying various dosage regimens [65]. The FOR-DMD trial is evaluating an alternative intermittent corticosteroid dosing regimen, but while follow-up has been completed, results have yet to be published. Recent research also supports the early initiation of twice-weekly corticosteroids, even in infants as young as 30 months, which would represent a significant departure from current practice. Prednisone and deflazacort are the two corticosteroids currently utilized in the treatment of DMD [14].

##### Prednisone and Deflazacort

**Figure 10:**
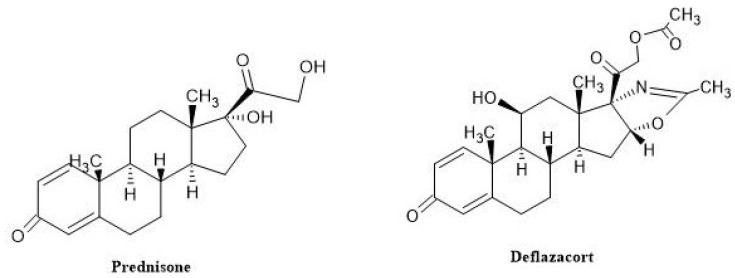
Illustration of the prednisone and deflazacort chemical structures.

Prednisone and deflazacort (Figure 10) are both heterocyclic compounds with four and five rings, respectively. Deflazacort exhibits distinct pharmacological properties compared to prednisone, including its reduced sodium-retaining capacity and lesser impact on glucose metabolism, which may be attributed to differences in their structural characteristics. Both prednisone and deflazacort influence various gene expression pathways, although the specific mechanisms of action in DMD remain uncertain. In a study examining gene expression in whole blood, researchers compared 14 children and adolescents with DMD receiving corticosteroid treatment to 20 untreated individuals. They found that corticosteroid treatment increased the expression of genes related to neutrophil granules, iron transport, and chondroitin sulfate synthesis. When researchers analyzed the gene expression of people treated with deflazacort with those treated with prednisone, they discovered that deflazacort caused fewer alterations in gene expression than prednisone did. These differences were observed in genes related to adipose metabolism. These findings suggest possible mechanisms for the differences in weight gain between the two treatments [66]. Since they have been in use for more than 20 years, the advantages are now widely recognized. They are the sole drugs that have been proven to boost muscle strength. Early research showed that their use increased functionality in daily tasks and sustained ambulation. Early prednisone clinical trials showed a definite advantage over natural history. Patients with DMD in the low- and high-dose groups of a double-blind, randomized clinical trial experienced improvements in muscular strength and function. Studies conducted afterward verified the improvements over time for pulmonary function and ambulation [67]. In 440 DMD patients who were enrolled in prospective, multi-center cohort research and followed for ten years (NCT-00468832), steroid therapy increased upper and lower extremity muscular strength in patients of all ages and increased life expectancy. The American Academy of Neurology (AAN) and a Cochrane Review both approved glucocorticoids for the treatment of DMD patients in 2016, although no exact dose guidelines were provided [68,69]. Although steroids enhance DMD patient outcomes, it is yet unknown which medication is best: prednisone or deflazacort. In a multi-center, double-blind, randomized study involving 18 participants, prednisone (0.75 mg/kg daily) and deflazacort (0.9 mg/kg daily) were compared over 12 months. Similar muscle improvements were observed with both steroids, with deflazacort resulting in less weight gain compared to prednisone. This study utilized a natural history cohort as a control group of individuals naïve to steroid treatment. A larger study involving 340 boys with DMD revealed that boys treated with deflazacort retained ambulation longer than those treated with prednisone. Nonetheless, this was linked with increased adverse effects, including short stature, a Cushingoid appearance, and cataracts.

In the FOR-DMD study, 196 DMD boys were randomly assigned to receive one of three daily regimens: deflazacort at 0.9 mg/kg or 1.2 mg/kg, or prednisone at 0.75 mg/kg. All three regimens resulted in comparable gains in muscle mass, although deflazacort was associated with a lower rate of weight gain and fewer behavioral side effects compared to prednisone. The clinical trial number for this investigation is NCT-01603407. A post hoc analysis from the DMD ACT trial’s placebo arm (ataluren) revealed that patients treated with deflazacort demonstrated better six-minute walk lengths and shorter times for climbing stairs compared to those treated with prednisone [70]. In a post hoc study involving the placebo groups of the Phase 3 ataluren trial and tadalafil studies, comprising 231 patients, deflazacort demonstrated a superior improvement in the 6 min walking distance (6MWD) and rise-from-supine time compared to prednisone. However, there were no significant differences observed in the 10 m run and North Star Ambulatory Assessment (NSAA) scores between the groups treated with steroids. A retrospective study involving 330 patients conducted for 13 years revealed that individuals receiving deflazacort maintained ambulation for an average of 15.6 years, whereas those receiving prednisone ambulated for an average of 13.5 years. The cause of this longer benefit is uncertain and could be attributed to factors such as the type of steroid or the earlier average age at the start of treatment (6.5 years for deflazacort vs. 8.1 years for prednisone). Furthermore, deflazacort was reported to increase lean body mass, reduce weight gain, and minimize the risk of scoliosis [70,71].

There is research that suggests deflazacort and prednisone may affect behavior differently. According to a study using questionnaires from 67 ambulant patients and their parents, those taking deflazacort acted more withdrawn than those taking prednisone/prednisolone, while those taking the latter drug acted more aggressively. It is difficult to assess safety and efficacy results due to the different corticosteroid doses used in prospective natural history studies conducted in the real world. However, DMD care recommendations do not mandate gradual weight-based dosage modifications for deflazacort and prednisone. These prospective natural history studies’ empirical data shed light on how these corticosteroids stack up against the actual clinical practice [66]. There are also some variations in the effectiveness and adverse effect profiles, despite the fact that both drugs have been found to restore the function of muscles in DMD patients. Individualized drug selection should be made according to the patient’s unique requirements and factors, and regular side effect monitoring is crucial in all situations.

#### 2.2.2. NF-kB Inhibition

Finally, due to the advantages of corticosteroids in DMD, preliminary studies to specifically stimulate NF-kB, one of its receptors, are also in progress. It is well established that NF-kB, a highly conserved protein complex, is a primary intracellular target of endocytosed glucocorticoid receptors and that it controls the transcription of genes involved in both healthy and degenerative aspects of DMD. Two present investigations, one on Vamorolone, a synthetic steroid, and another on edasalonexent, a nonsteroidal drug, are being examined for the efficacy of NF-kB inhibition in the management of DMD [64].

##### Vamorolone

**Figure 11:**
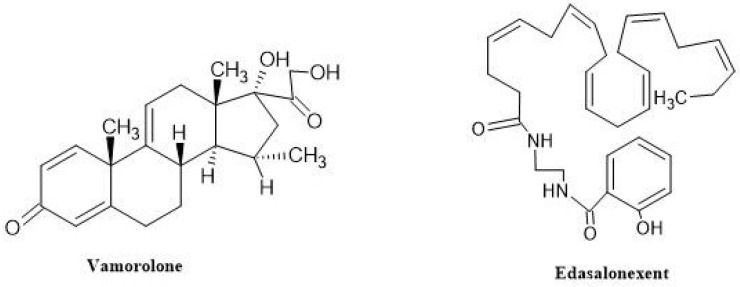
Vamorolone and edasalonexent structure.

Vamorolone (Figure 11) represents a novel drug that shows promise in surpassing the effects of traditional steroidal anti-inflammatory drugs. It works by restoring the physiochemical properties associated with membrane stabilization and inhibiting transrepression, which involves the NF-kB-mediated inhibition of anti-inflammatory activity. Moreover, vamorolone modulates mineralocorticoid receptor signaling, shifting it from an agonist to an antagonist, and does not engage in transactivation, which is the process of gene transcription via GRE-mediated binding of ligand/receptor dimers. Research conducted on vamorolone in animal models of DMD inflammation has demonstrated that it maintains anti-inflammatory action with fewer negative effects than prednisolone in chronic inflammatory situations.

In contrast to the conventional molecular models of ligand/receptor dimeric complexes, vamorolone works by blocking signals associated with NF-kB that trigger inflammation in a form that remains single. This property allows it to maintain its anti-inflammatory effects while reducing adverse reactions in preclinical models. During a 2-week treatment period, vamorolone showed improved safety profiles compared to traditional glucocorticoids, as assessed by blood biomarkers. Initial studies involved healthy adult males, followed by a 2-week treatment phase and a 2-week break. Subsequently, a Phase 2a study lasting 4 weeks was conducted in individuals diagnosed with DMD. The pharmacokinetics and metabolism of vamorolone are comparable to those of corticosteroids, and it is taken orally once daily [72]. All individuals who took part in the VBP15-002 trial were later included in a 24-week extended phase, designated as VBP15-003, receiving the same dosage. This extension was designed to fulfill the intent-to-treat criteria for pediatric clinical trials. A total of 48 boys diagnosed with DMD, aged 4 to 7 years and previously untreated with steroids, were involved in this open-label, multiple-ascending-dose investigation of vamorolone. The dose levels in an oral suspension formulation ranged from 0.25 to 6.0 mg/kg/d, with 12 boys allocated to each dose level. The main objective of this study was to establish the ideal vamorolone dosages. Over the course of the 24-week treatment period, oral administration of vamorolone at all tested dosages was safe and well tolerated. The primary efficacy outcome of enhanced muscular function was achieved by the 2.0 mg/kg/d dosage group without evidence of the majority of glucocorticoid side effects. In boys receiving vamorolone, osteocalcin, a marker of bone formation, was elevated, potentially indicating a reduction in the bone morbidities associated with glucocorticoids. In comparison with previously reported studies of glucocorticoid therapy, vamorolone-treated patients with DMD had lower biomarker outcomes for insulin resistance and adrenal suppression [73].

Further, the results of open-label vamorolone treatment after 30 months (NCT03038399) can be looked into. The Cooperative International Neuromuscular Research Group (CINRG) conducted a nonrandomized controlled experiment at 11 US and international study locations. During the 6-month dose-finding investigation, 46 boys with DMD, aged 4.5 to 7.5 years, participated. Data analysis was conducted between July 2020 and November 2021. These participants were then included in a long-term extension (LTE) study lasting 24 months, during which they received vamorolone at doses of 2.0 or 6.0 mg/kg/d. The primary outcome measure was the change in time-to-stand (TTSTAND) velocity from the baseline of the dose-finding study to the completion of the LTE study. The efficacy of the interventions was evaluated using the North Star Ambulatory Assessment (NSAA), the 6 min walk test, and timed function tests. DMD patients treated with vamorolone in the LTE trial were compared and contrasted with those treated with glucocorticoids in the Duchenne Natural History trial (DNHS) and the NorthStar United Kingdom (NSUK) Network. Out of the 46 DMD boys who completed the dose-finding study, approximately 41 (mean standard deviation age, 5.33 ± 0.96 years) also completed the LTE study. Among the participants receiving an increased dosage of vamorolone (2.0 mg/kg/d), the mean ± standard deviation TTSTAND velocity consistently decreased from baseline to 30 months (0.206 ± 0.070 rises/s vs. 0.189 ± 0.124 rises/s). However, this change did not show statistical significance (−0.011 rises/s; CI, −0.068 to 0.046 rises/s). Vamorolone was well tolerated at doses up to 6.0 mg/kg/d, with only 5 of the 46 patients stopping the study early and for unrelated causes. Glucocorticoid-treated DNHS individuals experienced a significant growth delay compared to vamorolone-treated participants, whose height percentiles remained consistent throughout time (0.37 percentile/month; 95% CI, 0.23 to 0.52 percentile/month) [74]. Once vamorolone completes its clinical trials successfully, it will be available to serve DMD patients.

##### Edasalonexent

Edasalonexent (CAT-1004) (Figure 11) was developed to inhibit NF-kB, which may slow the disease’s course by enhancing muscle function, preventing muscle aging, encouraging muscle regeneration, and lowering fibrosis and inflammation. It is a brand-new, orally administered small molecule that covalently joins two bioactive molecules that inhibit NF-kB, salicylic acid and docosahexaenoic acid (DHA, an omega-3 fatty acid). When given concurrently, salicylic acid and DHA have the ability to synergistically reduce NF-kB despite having distinct PK profiles, absorption and distribution properties, and mechanisms of action. The two bioactive molecules are covalently linked to make edasalonexent stable extracellularly. As soon as it enters the cells, edasalonexent is hydrolyzed by the enzyme fatty acid amide hydrolase (FAAH), which increases its effectiveness and simultaneously releases salicylic acid and DHA [75].

MoveDMD is a three-part study (NCT02439216). In the Phase 1 trial, males between the ages of 4 and 8 years who had been diagnosed with DMD based on their clinical phenotype and any DMD mutation were eligible to participate if they could walk on their own, with or without the aid of support, and if they had not taken any corticosteroids in the six months before to the screening. Following one week of treatment, the initial trial of edasalonexent in young DMD patients showed promising results, indicating good tolerability and suppression of NF-kB pathways. These encouraging findings support the chosen dosing regimen and warranted further clinical investigation of edasalonexent in Part B, which involved a 12-week double-blind, placebo-controlled study in paediatric DMD patients. Part B consisted of a 12-week, Phase 2 placebo-controlled research using edasalonexent at doses of 67 or 100 mg/kg to evaluate effectiveness, safety, and tolerability. Patients from Part B proceeded to an open-label extension study (Part C) after 12 weeks. To meet the target enrollment, new patients were recruited in addition to the Part A patients who were qualified to continue in Part B. The MoveDMD study’s Part B and extension phase (Part C) leveraged the off-treatment period that patients who completed Part A of the trial had before initiating Part B, typically lasting 30 weeks. Following a 14-day eligibility period and baseline screening, recruited patients were randomly assigned in a 1:1:1 ratio to receive either oral placebo, 67 mg/kg/day (administered in two divided doses), or 100 mg/kg/day (administered in three divided doses) of edasalonexent for 12 weeks. Stratified Randomization was conducted according to baseline age and the duration needed to complete the 10 m walk/run test (10MWT). Patients assigned to the placebo group were additionally randomized in a 1:1 ratio to receive a placebo in either two or three divided doses to ensure alignment with medication allocation. Following the 12-week double-blind study, patients received open-label edasalonexent for at least 72 weeks, at doses of 67 mg/kg/day or 100 mg/kg/day. An additional protocol modification extended the study duration to 150 weeks. Given the positive results observed in muscle function tests among participants receiving 100 mg/kg and the favorable safety profile, the protocol was amended to raise the dosage of the 67 mg/kg group to 100 mg/kg during the open-label Part C extension. The data collected from these phases were utilized to design a Phase 3 trial.

The multinational, Phase 3, placebo-controlled trial with randomization at a ratio of 2:1 (PolarisDMD) research looked at the impact of edasalonexent (100 mg/kg/day) versus a placebo over a 52-week period in patients with DMD who were 4 to 8 years old and had any type of dystrophin mutation. Between November 2018 and September 2020, the PolarisDMD (NCT03703882) study was carried out at 37 locations throughout eight nations (the US, Canada, UK, Ireland, Germany, Sweden, Israel, and Australia). In this double-blind trial, participants were enrolled and randomized to receive edasalonexent softgel capsules or matched placebo softgel capsules every day for 52 weeks. Patients were administered edasalonexent orally in three divided doses, approximately 33 mg/kg each, amounting to a total daily dosage of 100 mg/kg. The number of pills needed for each patient was calculated based on their weight and inputted into the interactive response web system (IRWS) [76]. This ensured accurate administration of the medication according to each patient’s individual dosage requirements. To increase absorption, the capsules were taken orally with food that included at least 8 g of fat. Age at baseline, time to stand up from a seated position, eteplirsen treatment, and area were used to stratify the randomization. Siblings of patients who had been randomly assigned to a therapy group were also eligible to take part in the trial; however, in order to prevent patients from the same household obtaining different treatment allocations, they were not randomized and were instead assigned to the same group. After the study ended, patients were eligible to enroll in an open-label extension trial. Because steroid treatment was not stopped for the experiment, boys in the trial were those for whom the administration of corticosteroids was either not yet appropriate or delayed by a decision made by their parents or guardians. To mitigate risks to participants, the study implemented enrollment criteria aimed at excluding boys who showed clear signs of decline.

Rather, its focus was on enrolling boys who were either unsuitable candidates for steroid treatment or whose parents opposed such treatment. This approach aimed to prioritize the safety and well-being of the participants while ensuring that the study’s objectives could be effectively met. Every three months, researchers were made to evaluate patients’ clinical trajectories and recommend starting steroids if they believed that the clinical progression called for discontinuation. A separate Data Safety Monitoring Board also examined the open safety and efficacy data every six months. Thus, in this study, 131 patients who received either edasalonexent or a placebo for 52 weeks revealed no statistically significant differences in the NSAA total score or TFTs between the two groups, despite the edasalonexent group consistently displaying reduced functional decline. However, a predetermined analysis by age revealed that for various measurements, edasalonexent and placebo exhibited greater significant differences in younger individuals (6.0 years). The majority of side effects were modest and mostly affected the gastrointestinal tract, and the medication was well tolerated. Edasalonexent was usually well tolerated and safe at a dose of 100 mg/kg/day, and although it did not significantly improve primary and secondary functional endpoints, it may decrease the course of the disease if started before six years old [77].

#### 2.2.3. Dysregulation of Calcium

Due to membrane rips and faulty Ca^2+^ release channels caused by dystrophin deficiency, the intracellular Ca^2+^ equilibrium is compromised, which causes chronic inflammation, regenerative cycles, and fibrosis. In the myofibers of DMD patients, high Ca^2+^ levels are seen during both rest and activity. By encouraging Ca^2+^-dependent proteinase to break down intracellular proteins, high cytosolic Ca^2+^ can exacerbate the dystrophic disease. Ca^2+^ channels can be indefinitely blocked by streptomycin. In mdx mice, long-term streptomycin therapy can improve limb muscle pathology by reducing fibrosis, boosting sarcolemmal stability, and encouraging muscle regeneration. However, treatment with streptomycin has no beneficial effects on the heart muscle and diaphragm [78]. A peptide called AT-300 (Akashi Therapeutics) inhibits mechanosensitive Ca^2+^ channels, providing very minor advantages in mdx mice. Clinical study preparations are now underway. Dystrophic muscles experience dissociation of the intracellular calcium calstabin-RyR channel complex as a result of post-translational alterations of the ryanodine receptor subtype 1 (RyR1). The small molecule ARM210/S48168 (ARMGO Pharma) acts as a stabilizer and treatment with it improves functional outcomes in mdx mice, particularly in their diaphragms (Capogrosso et al., abstract in Neuromuscul Disord. G.P.90 2014, 24:9–10) [47]. The transmembrane protein known as the Na^+^-H^+^ exchanger 1 (NHE1/Rimeporide) controls intracellular Ca^2+^ levels in addition to controlling the volume of Na^+^ and H^+^ in cells. Both DMD patients and mdx mice have higher intracellular Na^+^ concentrations in their skeletal muscles. Muscular edema is a side effect of intracellular Na^+^ excess in muscles and contributes to muscular deterioration. Inhibiting NHE-1, Rimeporide has been shown to have anti-inflammatory and anti-fibrotic actions in mdx animals. In Phase 1b research of Rimeporide, the beneficial effects on a number of pharmacodynamic biomarkers, including IGFBP1 and IGFBP6, have been revealed [79]. Repairing the plasma membrane serves as an alternative strategy, involving the process of healing cell membranes. Mitsugumin 53 (MG53), a vital component specific to muscles, shields cardiac and skeletal muscle cells from various acute injuries and prolonged physiological pressures [27].

#### 2.2.4. Targeting Histone Deacetylases

Delocalization and downregulation of nNOS result in inadequate S-nitrosylation and constitutive stimulation of Histone deacetylase 2 (HDAC) in dystrophin-deficient muscles. HDAC facilitates the removal of acetyl residues from lysine in both histone and nonhistone proteins. Muscle regeneration may be hampered and microfiber response to contraction may be compromised by disrupted DAPC-NO (Dystrophin-Associated Protein Complex-Nitric Oxide) signaling and hyperactive HDAC. Therefore, methods using HDAC inhibitors (HDACis) to reduce increased HDAC activity among dystrophic muscles are an intriguing direction [47]. Dystrophic muscles were found to have increased HDAC activity, and mdx mice were used to show that HDACis are efficacious. The mechanism by which HDACis function may involve a rise in the concentration of follistatin, a key regulator of muscle regeneration. Follistatin is recognized for its role in decreasing the levels of myostatin, a protein that inhibits muscle fiber regeneration, as discussed in “Dystrophin-independent gene therapies.” However, it is worth noting that HDACis might also exert their effects via pathways distinct from the myostatin signaling cascade. This suggests a multifaceted mechanism of action for HDACis in promoting muscle regeneration and function [80]. Early investigations focused on evaluating three structurally unrelated HDAC medications, phenylbutyrate (PhB), valproic acid (VPA), and trichostatin A (TSA), all of which were already in clinical use for various therapeutic purposes. Among these, TSA emerged as the primary agent responsible for restoring both muscle function and morphology in dystrophic animals. Additionally, suberoylanilide hydroxamic acid (SAHA), another HDACi, has been studied in mdx mice. Administered to mdx mice over three months at doses ranging from 0.6 to 5 mg/kg/day, SAHA proved effective in slowing the progression of the disease. These findings underscore the potential therapeutic benefits of HDAC inhibitors in treating muscular dystrophy [81]. The extensive research conducted on the HDAC-inhibiting drug givinostat culminated in the initiation of Phase 3 clinical trials (NCT03373968, NCT02851797). Early studies on dystrophic mice revealed promising results, with givinostat demonstrating efficacy comparable to TSA in this condition. Administered at doses of 5 and 10 mg/kg/day, givinostat significantly improved muscle function, as evidenced by fatigue treadmill tests, while reducing fibrotic scarring, fatty infiltration, and inflammation in the muscle tissue of mdx mice. These compelling findings prompted the initiation of a Phase 1 clinical trial in January 2013 (NCT01761292), underscoring the potential therapeutic prospects of givinostat for humans [82]. According to Bettica et al., givinostat was administered to 20 boys with DMD aged 7 to 10 years for approximately 12 months, ranging from 25 mg BID to 37.5 mg BID, based on the regimen, all of whom were receiving stable corticosteroid therapy. Although the trial’s small sample size may have limited the ability to observe functional improvements, histological analysis of muscle biopsies revealed promising outcomes, including increased muscle fiber size, decreased fibrosis, reduced tissue necrosis, and replacement of fatty tissue. Notably, the European Medicines Agency (EMA) has granted orphan drug designation (EU/3/12/1009) to givinostat for the treatment of DMD, highlighting its potential therapeutic significance. Currently, Phase 3 trials for the drug are underway, with an estimated enrollment of 313 individuals (100 for NCT03373968 and 213 for NCT02851797). These trials are expected to provide further insights into the efficacy of HDAC inhibitors and their potential for extended treatment in patients with DMD [83].

#### 2.2.5. Redox Imbalance

One of the characteristics of dystrophic muscles is oxidative stress. Increased oxidative stress is the result of Ca^2+^ overload, which also increases the formation of reactive oxygen species (ROS). Ca^2+^-induced dysfunction of mitochondria and necrosis are made worse by ROS causing inflammation through NF-B and TGF pathway activation [84]. Preliminary investigations into Raxone/idebenone, a synthetic variant of Coenzyme Q10, were inspired by initial findings from studies involving boys with DMD treated with the hydrophobic antioxidant Coenzyme Q10. Coenzyme Q10 operates by binding to the inner mitochondrial membrane, where it functions as an electron acceptor for complexes I (NADH) and II (SDH) of the respiratory chain. These early results laid the groundwork for further exploration of the potential benefits of idebenone in the context of DMD treatment [85]. It has been demonstrated that this antioxidant from Santhera Pharmaceuticals, which encourages mitochondrial electron reflux, is cardioprotective and improves the exercise capacity of mdx mice. Patients with DMD experienced less loss of respiratory function, according to a recent Phase 3 clinical trial (NCT01027884) [86]. Other antioxidant treatments are being researched right now, including melatonin and N-acetylcysteine. Simvastatin recently demonstrated excellent promise in mdx mice [87]. Simvastatin operates by lowering LDL cholesterol levels and mitigating oxidative stress through the decreasing of nicotinamide adenine dinucleotide phosphate-oxidase 2 (NOX2), a pivotal contributor to reactive oxygen species (ROS) production. This medication is a common statin prescribed for children; however, itis important to keep in mind that statin use has adverse effects that can affect the muscles and that this drug has been repurposed and is currently being investigated for DMD. An inherently occurring antioxidant is N-acetylcysteine (NAC). Given that NAC injection has been shown in multiple pre-clinical investigations to bring down the dystrophic pathology in both skeletal and cardiac muscles of mdx mice, it may be a viable therapy option for DMD males. NAC therapy, however, significantly reduces body weight increase and muscle mass in mdx mice. Therefore, when applying NAC clinically in the future, these potential side effects must be given more thought [88].

Due to mitochondrial failure caused by overly increased intracellular Ca^2+^ in DMD, Ca^2+^ buffering from myofibers and organelles is impaired, which lowers ATP generation and raises ROS. Large mitochondrial permeability transition pores (MPTPs), which are mediated by cyclophilin D, also result in the permeabilization of mitochondria. Alisporovir/Debio-25, a cyclosporine analogue that has been demonstrated to block cyclophilin D and stop MPTP synthesis, was created to address this flaw. A reduction in inflammation and macrophage infiltration in mdx mice was more effectively accomplished by treatment with this drug than by prednisone [89]. An alternative strategy is the use of epicatechin (Cardero Therapeutics), a kind of flavonoid released in response to physical activity. Epicatechin uses the NO/AMPK/SIRT1/PGC-1 pathway to cause an oxidative phenotype, which mimics the effects of specific exercise regimens and leads to mitochondrial biogenesis, a decrease in oxidative stress, and a rise in utrophin and follistatin (NCT01856868).

#### 2.2.6. Muscle Hypoxia, Cardiac Impairment, and Muscle Atrophy

The absence of dystrophin in DGC complex components like nNOS results in the misplacement of nNOS in the sarcolemma, resulting in decreased levels of nNOS mRNA and protein, diminished nNOS activity, and reduced nitric oxide (NO) production. This imbalance affects muscle oxygenation during muscle use, weakens defenses against excessive sympathetic vasoconstriction, and induces active muscular hypoxia. Consequently, soluble guanylate cyclase (sGC) activity declines due to decreased nNOS activity, leading to a reduction in cyclic guanosine monophosphate (cGMP) levels. In the realm of DMD treatment, substances that inhibit cGMP phosphodiesterase (PDE5) activity and prolong the biological half-life of cGMP hold particular significance. Notably, two PDE5 inhibitors, tadalafil (Cialis^®^) and sildenafil (Viagra^®^), have demonstrated substantial improvements in mdx mice, offering potential therapeutic avenues for DMD [90,91]. These data suggest that, despite the lack of clear mechanistic explanations, PDE5 inhibitors may be useful therapeutic approaches for DMD. Although the PDE inhibitor was given in two different regimens, a bigger study with over 300 children with DMD who were divided into groups, treated with tadalafil and control, revealed no difference between the PDE inhibitor and boys who received a placebo (who were all taking standard GCs). Despite the thorough supervision of this Phase 3 study, which investigated the potential functional improvement resulting from the daily administration of a PDE5 inhibitor over 48 weeks, no evidence was found to support the protective effects of the inhibitor (NCT01865084). Additionally, the corresponding study for tadalafil was discontinued due to ineffectiveness. In a clinical investigation, the other inhibitor, sildenafil, failed to significantly enhance heart function. Aside from that, the investigation of sildenafil was stopped due to the increased number of negative effects [92,93]. Tadalafil may have a good effect on heart and leg muscle injuries, but more research is required to thoroughly examine this possibility. This is because several factors, such as patient age and exercise regimen, may have an impact on the results. Loss of dystrophin in DMD also results in decreased NO synthase (NO) activity. Intramuscular L-arginine can convert to NO in healthy muscle tissue. The bioavailability of NO is decreased in DMD patients due to higher production of uneven dimethylarginine (ADMA) and decreased synthesis of homoarginine (hArg) [94]. Elevating L-arginine levels presents a positive approach to enhance mitochondrial function and mitigate the inherent oxidative stress associated with DMD. Studies conducted on mdx mice have shown that supplementing with L-arginine can alleviate inflammation and promote muscle regeneration, indicating its potential therapeutic benefits in the context of DMD management. Additionally, a Phase 3 trial revealed that giving DMD patients L-arginine and metformin together delayed the loss of muscular function [95]. While pharmacological therapies have demonstrated promising results in managing DMD, there remains uncertainty regarding the necessity of preventive cardiac therapy for all DMD patients and the optimal combination of cardioprotective drugs. Additional investigation is needed to identify the most effective approach to cardiac care in DMD, including identifying which patients would benefit most from preventive treatment and determining the most suitable combination of cardioprotective medications [63].

Therapies that focus on the heart may be beneficial for DMD patients. Because angiotensin II works as a destructor, encouraging oxidative stress, cardiac fibrosis, and cardiomyocyte death in DMD patients, angiotensin-converting enzyme inhibitors (ACEIs) may have cardioprotective effects. A 10-year follow-up study found that presymptomatic perindopril (ACEI) medication taken for three years resulted in a considerably greater survival rate [96]. Starting ACEI medication even before the beginning of LV dysfunction is advised. As an alternative to ACEIs, angiotensin receptor blockers (such as losartan) can be used to reduce the adverse consequences of angiotensin in cardiac failure [97]. Beta-adrenergic receptor (β-AR) blockers serve as supplementary medications commonly employed as second-line treatment in DMD patients who are exhibiting symptoms of left ventricular (LV) dysfunction. Mineralocorticoid receptor (MR) antagonists, including eplerenone, have also been taken into consideration because of their dual cardioprotective and anti-fibrotic qualities [98].

The control of muscle protein production and breakdown is crucial for maintaining skeletal muscle mass. In muscular damage models, 2-agonists have been shown to improve lean body mass, promote muscle protein synthesis, and start satellite cell proliferation. A 2-agonist called DT-200 was initially studied as a treatment for cachexia and sarcopenia. DT-200 dramatically lessens muscular atrophy in the hindlimb and enhances running efficiency in mdx mice. Trials on individuals with DMD have not yet started, though [99]. Myostatin is a secretory protein that is unique to muscles and belongs to the TGF superfamily. It inhibits the creation of new muscle. Myostatin reduces the level of expression of transcription factors necessary for cell proliferation in myogenic cells and prevents myoblast development. Myostatin suppression is a viable strategy for treating DMD as a result. Myostatin’s inactivation caused a noticeable increase in muscle mass in mdx [100]. Myostatin-binding protein folistatin (FST) can promote muscle growth by reducing inflammation brought on by myostatin. When FST was delivered by an AAV in mdx animals, the muscular pathology was reversed, and the animals’ strength increased [80]. An isoform of FST called folistatin-344 has a positive impact on muscle development and performance. rAAV1.CMV.huFollistatin344 is currently the subject of a Phase 1/2 investigation in DMD patients (NCT02354781) [101]. Urocortins (Ucns), a type of neuropeptide, are intricately linked to the hypothalamic hormone corticotropin-releasing factor (CRF) [102]. Two Ucns receptors, CRF1R and CRF2R, strongly link to G-proteins to stimulate adenylyl cyclase and encourage the production of cAMP. The diaphragm can be protected from deterioration and have some of its force lost by activating the CRF2R and upregulating cAMP downstream [103]. Additional research in mdx mice showed that Ucns enhanced the shape and function of dystrophic muscles, suggesting that Ucns may have considerable potential as a means of treatment for DMD [104].

Based on the preceding discussion, the molecules that are currently in clinical trials or have been approved are listed in Table 1.

## 3. Discussion and Conclusions

As a whole, this in-depth review offers a broad perspective on the diverse array of therapeutic approaches used to treat DMD. This overview highlights the unrelenting search for a DMD cure, from the ground-breaking developments in exon skipping therapies to the exciting potential of AAV-mediated gene therapies. A further benefit of secondary therapies is that they provide hope for individuals, supporting the tailored approach to DMD medicines. The inclusion of a thorough table describing active DMD clinical trials highlights the changing environment of experimental therapies and is a useful resource for doctors, researchers, and patients.

These approaches, which range from cutting-edge genetic medicines to newly discovered small molecules, provide a ray of hope for patients and their families and highlight the scientific community’s concerted efforts to tackle this debilitating illness. Despite the difficulties still present, the advances made in DMD research give hope for a better future.

## Figures and Tables

**Table 1:** An extensive overview of DMD clinical trials.

Title	Current Stage	Type	Clinical Trial ID	Company
Exon Skipping Therapy				
Casimersen	Phase 2 (Approved)	Antisense Oligonucleotides	NCT04179409	Sarepta Therapeutics, Inc.
Eteplirsen				
Golodirsen				
Viltolarsen	Phase 3 (Approved)	Antisense Oligonucleotides	NCT04060199	Nippon Shinyaku Co., Ltd.
Stop codon Readthrough				
Gentamicin	Phase 1 (Completed)	Small molecule	NCT00451074	Nationwide Children’s Hospital
Ataluren	Phase 3 (Completed)	Small molecule	NCT01557400	PTC Therapeutics
NPC-14 (Arbekacin Sulfate)	Phase 2	Small molecule	NCT01918384	Japan Medical Association Nobelpharma
AAV-mediated gene transfer				
rAAV2.5-CMV-minidystrophin (d3990)	Phase 1 (Completed)	Injectable viral vector	NCT00428935	Asklepios Biopharmaceutical, Inc.
scAAV9.U7.ACCA	Phase 1/2 (Active)	Injectable viral vector	NCT04240314	Audentes Therapeutics
rAAV1.CMV.huFollistin344	Phase 1/2 (Completed)	Injectable viral vector	NCT02354781	Duchenne Alliance Milo Therapeutics
SGT-001	Phase 1/2 (Active)	Injectable viral vector	NCT03368742	Solid Biosciences Inc.
RGX-202	Phase 1/2 (Active)	Injectable viral vector	NCT05693142	REGENXBIO Inc.
PF-06939926	Phase 1 (Active)	Injectable viral vector	NCT03362502	Pfizer
Delandistrogene Moxeparvovec	Phase 3 (Completed and Approved)	Injectable viral vector	NCT05096221	Sarepta Therapeutics, Inc.
Increasing utrophin levels (mention in test that it is normally expressed in fetus)				
Ezutromid	Phase 2	Small molecule	NCT02858362	Summit Therapeutics
(−)-epicatechin	Phase 1/2	Small molecule	NCT01856868	Cardero Therapeutics, Inc.
rAAVrh74.MCK.GALGT2	Phase 1/2 (Active)	Injectable viral vector	NCT03333590	Kevin Flanigan, Nationwide Children’s Hospital
Post-transcriptional gene silencing				
RO7239361	Phase 2/3 (Completed)	Antisense Oligonucleotides	NCT03039686	Hoffmann-La Roche
PF-06252616	Phase 2 (Terminated)	Monoclonal Antibody	NCT02907619	Pizer
Glucocorticoids				
Prednisone	Phase 2 (Active)	Small molecule	NCT04322357	U.S. Army Medical Research and Development Command
Tamoxifen	Phase 3 (Completed)	Selective estrogen receptor modulator (SERM)	NCT03354039	University Hospital, Basel, Switzerland
Deflazacort	Approved	Small molecule	NCT02592941	PTC Therapeutics
NF-kB inhibitors				
Edasalonexent	Phase 3	Small molecule	NCT03703882	Catabasis Pharmaceuticals
Vamorolone	Phase 2 (Active)	Small molecule	NCT05185622	Santhera Pharmaceuticals
Dysregulation of calcium				
Rimeporide	Phase 1 (Completed)	Small molecule	NCT02710591	EspeRare Foundation
AT-300	Pre-clinicals	Small molecule	-	Akashi Therapeutics
HDAC				
Givinostat	Approved	Small molecule	NCT02851797	Syneos Health
Redox imbalance				
Coenzyme Q10	Phase 2 (Completed)	Small molecule	NCT00033189	Cooperative International Neuromuscular Research Group
Idebenone	Phase 3 (Terminated)	Small molecule	NCT03603288	Santhera Pharmaceuticals
Muscle atrophy, Cardiac impairment, and Muscle hypoxia				
Tadalafil	Phase 3 (Terminated)	Small molecule	NCT01865084	Eli Lilly and Company
Sildenafil	Early Phase 1 (Completed)	Small molecule	NCT01359670	Cedars-Sinai Medical Center
L-arginine	Phase 1 (Completed)	Small molecule	NCT01388764	Massachusetts General Hospital
Metformin	Phase 1 (Completed)	Small molecule	NCT02516085	University Hospital, Basel, Switzerland

## Data Availability

Not applicable.
